# RETRACTED: Sydykov et al. Genetic Deficiency and Pharmacological Stabilization of Mast Cells Ameliorate Pressure Overload-Induced Maladaptive Right Ventricular Remodeling in Mice. *Int. J. Mol. Sci.* 2020, *21*, 9099

**DOI:** 10.3390/ijms27198688

**Published:** 2026-09-29

**Authors:** Akylbek Sydykov, Himal Luitel, Argen Mamazhakypov, Malgorzata Wygrecka, Kabita Pradhan, Oleg Pak, Aleksandar Petrovic, Baktybek Kojonazarov, Norbert Weissmann, Werner Seeger, Friedrich Grimminger, Hossein Ardeschir Ghofrani, Djuro Kosanovic, Ralph Theo Schermuly

**Affiliations:** 1 Excellence Cluster Cardio-Pulmonary Institute (CPI), Universities of Giessen and Marburg Lung Center (UGMLC), Member of the German Center for Lung Research (DZL), Justus Liebig University of Giessen, Aulweg 130, 35392 Giessen, Germany; 2 Veterinary Science (Theriogenology), Center for Biotechnology, Agriculture and Forestry University (AFU), Rampur 44209, Chitwan, Nepal; 3 Department of Lung Development and Remodelling, Max-Planck Institute for Heart and Lung Research, Parkstrasse 1, 61231 Bad Nauheim, Germany; 4 Department of Biochemistry, Universities of Giessen and Marburg Lung Center, Justus Liebig University of Giessen, Friedrichstrasse 24, 35392 Giessen, Germany; 5 Department of Pulmonology, Sechenov First Moscow State Medical University (Sechenov University), 119992 Moscow, Russia

The journal retracts the article titled “Genetic Deficiency and Pharmacological Stabilization of Mast Cells Ameliorate Pressure Overload-Induced Maladaptive Right Ventricular Remodeling in Mice” [1], cited above.

Following publication, concerns were brought to the attention of the Editorial Office regarding the integrity of images presented in this publication [1].

Adhering to our standard procedure, the Editorial Office and Editorial Board conducted an investigation that confirmed inappropriate editing and duplications within Figures 2 and 5 presented in this article. Although the authors cooperated with the investigation, the explanations and supporting materials provided were not deemed sufficient by the Editorial Board to resolve the identified concerns.

As a consequence, the Editorial Board has lost confidence in the reliability of the reported findings and has decided to retract this publication in accordance with MDPI’s retraction policy.

This retraction was approved by the Editor-in-Chief of the journal *International Journal of Molecular Sciences*.

The authors have been informed of this retraction but did not provide a comment on this decision.
